# Impact of *PAX6*-Related Congenital Aniridia on Corneal Diameter, Central Corneal Thickness and Keratometry

**DOI:** 10.3390/jcm15051805

**Published:** 2026-02-27

**Authors:** Kitti Kormányos, Béla Csákány, Mária Csidey, Annamária Náray, Klaudia Kéki-Kovács, Orsolya Németh, Krisztina Knézy, Mária Bausz, Andrea Szigeti, Anita Csorba, Dorottya Szabó, Marta Corton, Kálmán Tory, Eszter Jávorszky, Zoltán Zsolt Nagy, Achim Langenbucher, Erika Maka, Nóra Szentmáry

**Affiliations:** 1 Department of Ophthalmology, Semmelweis University, 1085 Budapest, Hungary; kormanyos.kitti@semmelweis.hu (K.K.); csakany.bela@semmelweis.hu (B.C.); mcsidey@yahoo.com (M.C.); naray.annamaria@semmelweis.hu (A.N.); kovacs.klaudia@semmelweis.hu (K.K.-K.); knezy.krisztina@semmelweis.hu (K.K.); bausz.maria@semmelweis.hu (M.B.); szigeti.andrea@semmelweis.hu (A.S.); csorba.anita@semmelweis.hu (A.C.); dorka.szaboo@gmail.com (D.S.); nagy.zoltan.zsolt@semmelweis.hu (Z.Z.N.); maka.erika@semmelweis.hu (E.M.); 2 Heim Pál National Pediatric Institute, 1089 Budapest, Hungary; 3 Dr. Rolf M. Schwiete Center for Limbal Stem Cell and Congenital Aniridia Research, Saarland University, 66424 Homburg/Saar, Germany; 4 Department of Ophthalmology, Markusovszky University Teaching Hospital, 9700 Szombathely, Hungary; nemeth.orsolya22@gmail.com; 5 Department of Genetics and Genomics, Instituto de Investigación Sanitaria-Fundación Jiménez Díaz University Hospital (IIS-FJD), Universidad Autónoma de Madrid (UAM), 28049 Madrid, Spain; marta.corton@gmail.com; 6 Center for Biomedical Network Research on Rare Diseases (CIBERER), Instituto de Salud Carlos III, 28029 Madrid, Spain; 7 MTA-SE Lendület Nephrogenetic Laboratory, Hungarian Academy of Sciences, 1051 Budapest, Hungary; tory.kalman@semmelweis.hu; 8 1st Department of Pediatrics, Semmelweis University, 1085 Budapest, Hungary; javorszky.eszter@semmelweis.hu; 9 Department of Experimental Ophthalmology, Saarland University, 66424 Homburg/Saar, Germany; achim.langenbucher@uks.eu

**Keywords:** congenital aniridia, biometry, AL, CD, CCT, ACD, LT, K, lens, MOVU

## Abstract

**Background/Objectives**: *PAX6* haploinsufficiency-related congenital aniridia is a panocular disease affecting multiple ocular structures. The aim of this study was to determine the biometric properties of eyes affected by *PAX6* haploinsufficiency-related classical congenital aniridia using a non-contact device. **Methods**: Fifty-nine eyes from 31 aniridia patients (48.39% male; mean age 27.0 ± 17.65 years, range 7–56) and 99 eyes from 50 healthy controls (44.00% male; mean age 28.56 ± 21.73 years, range 4–81) were examined using the Movu biometer (Argos Inc.). Axial length (AL), corneal diameter (CD), central corneal thickness (CCT), anterior chamber depth (ACD), lens thickness (LT), pupil size (PS), and mean keratometric value (K-mean) were measured. **Results**: Linear mixed-effects models showed significant effects of diagnosis on CCT (β = 182.39, *p* < 0.001), CD (β = −0.55, *p* = 0.02), and K-mean (β = −1.10, *p* = 0.03), while axial length was associated with gender (β = −0.90, *p* = 0.03). Mann–Whitney testing showed no interocular asymmetry (all *p* ≥ 0.07; η^2^ ≤ 0.04) overall. **Conclusions**: *PAX6*-related congenital aniridia eyes are associated with increased CCT and reduced CD, and K-mean, while AL appears to be mainly influenced by gender. The absence of marked interocular asymmetry suggests relatively symmetrical bilateral involvement. These differences should be considered in corneal and lens surgery planning.

## 1. Introduction

Congenital aniridia is a rare panocular disease affecting different structures of the eye with varying severity. Its main features are partial or total absence of the iris, but it may also be associated with nystagmus, progressive aniridia-associated keratopathy (AAK), secondary glaucoma, lens aberrations (cataract, ectopia lentis), optic nerve head, and foveal hypoplasia [1,2,3]. Global prevalence of this rare disease is between 1/40,000 and 1/100,000 [4]. In 90% of the cases congenital aniridia is caused by a heterozygous mutation in the *PAX6* gene [5], which is a major regulator of eye, brain, and pancreas development. Vertebrates are highly susceptible to even less severe lesions of the *PAX6* gene and a homozygous *PAX6* mutation may result in anophthalmia and perinatal lethality [6].

In patients with congenital aniridia, AAK is the main reason for progressive visual loss, often affecting more than 80% of the patients [7]. Development and progression of AAK is also related to changes in PAX6 function. AAK progresses with age, resulting in progressive limbal stem cell deficiency, stromal scarring, neovascularization, corneal vascularized pannus formation, and conjunctivalization [8,9,10]. Over time, corneal surgeries such as penetrating keratoplasty or the combination of corneal transplantation with implantation of Boston Keratoprosthesis may be considered as treatment options for improving vision. Nevertheless, these procedures carry an increased postoperative risk of complications, including persistent epithelial defects, suture loosening, and graft rejection, classifying them as high-risk interventions. Moreover, long-term visual improvement is not guaranteed [11].

In patients with congenital aniridia, cataract prevalence ranges from 50 to 90% [12]. There may be congenital cataract (most typically anterior polar cataract), but in addition, juvenile cataract usually also develops in the first two decades of life (most typically with posterior cortical opacity) [7]. The Human *PAX6* Allelic Variant Database has identified more than 60 *PAX6* pathogenic variants that have been associated with congenital aniridia and congenital cataract [13,14,15]. Surgical management of these early-onset congenital cataracts is characterized by a higher surgical risk and limited postoperative visual recovery. In addition, postsurgical changes in the anterior segment, ranging from mild alterations to the development of the so-called aniridia fibrosis syndrome, may jeopardize postoperative visual outcomes [16].

In order to carefully plan any kind of surgery, exact measures of anatomical structures are extremely important to reduce the risk of complications and to achieve the best postoperative visual acuity possible. Besides the AAK and cataracts, there is often nystagmus and foveal/macular hypoplasia in these patients, which may also have an effect on the measurements [7]. Of course, foveal/macular and optic disk hypoplasia may reduce both preoperative and postoperative expected best-corrected visual acuity in congenital aniridia patients.

Ocular biometry may be performed using ultrasound, optical methods such as partial coherence interferometry, and using Fourier domain optical coherence tomography (OCT) [17]. The Argos Movu biometer (Argos Inc., Santa Clara, CA, USA) uses swept-source optical coherence tomography (SS-OCT) and belongs to Fourier domain OCT devices. This technology enables biometric measurements even with corneal scars, dense cataracts, or vitreous hemorrhages, through the good penetration of light [18].

To the best of our knowledge, there was no previous study to analyze biometric properties of a larger cohort of congenital aniridia subjects, compared to those in normal controls, using an SS-OCT biometer. The aim of the study was to determine axial length (AL), corneal diameter (CD), central corneal thickness (CCT), anterior chamber depth (ACD), lens thickness (LT), pupil size (PS), and keratometric data in congenital aniridia subjects, compared to healthy individuals.

## 2. Patients and Methods

This cross-sectional study adhered to the principles of the Declaration of Helsinki and was approved by the Semmelweis University Regional and Institutional Committee of Science and Research Ethics (ethical approval number SE-RKEB-80/2020). Participation in the study was voluntary, and written informed consent was obtained from all participants or legal guardians under 18 years of age.

In the congenital aniridia group, 59 eyes from 31 patients (48.39% male, mean age 27.17 ± 17.75 years [range: 7–61 years]) were enrolled. The participants were diagnosed and/or treated at the Department of Ophthalmology at Semmelweis University between 2005 and 2020. A total of 99 eyes from 50 randomly selected healthy individuals served as controls (44.0% male, mean age 28.56 ± 21.73 years [range: 4–81 years]). ANOVA showed no significant between-group differences in age, demonstrating appropriate age matching of controls (*p* = 0.67).

The 31 congenital aniridia patients belonged to 19 families. Among them, 7 patients (21.21%) from 3 families had a *PAX6* splicing mutation; 6 patients (18.18%) from 4 families had a *PAX6* nonsense mutation; 6 individuals (18.18%) from 6 families had a *PAX6* frameshift mutation; 5 patients (15.15%) from 1 family had a microdeletion affecting the 3′ regulatory region; 4 patients (12.12%) from 2 families had a *PAX6* microdeletion; and 2 individuals (6.06%) from 1 family had a *PAX6* missense mutation. In one patient (3.23%) from one family with classical aniridia, the genetic variation remains unidentified.

First, all participants underwent a detailed slit-lamp biomicroscopic examination to grade AAK and iris malformation according to the criteria established by Lagali et al. [19], and to document lens status (clear lens, cataract, pseudophakia, aphakia).

AAK severity was graded on a scale from 0 to 5. Grade 0 indicated the absence of limbal involvement. Grade 1 was defined by conjunctival tissue extending across the limbus but limited to less than 1 mm of corneal involvement. Grade 2 corresponded to a circumferential pannus affecting the peripheral cornea. Grade 3 was characterized by pannus progression into the central cornea, frequently accompanied by complete vascular coverage. Grade 4 denoted total corneal vascularization, while Grade 5 represented advanced disease with a thickened, opaque, and fully vascularized corneal surface [19].

Iris hypoplasia or malformation in patients with congenital aniridia was categorized into four grades based on slit-lamp examination without gonioscopy: atypical coloboma (Grade 1), iris remnants exceeding 6 clock hours (Grade 2), iris remnants involving fewer than 6 clock hours (Grade 3), and complete absence of visible iris tissue (Grade 4) [19]. All AAK and iris malformation assessments were conducted by a single examiner (N.S.).

Subsequently, biometric measurements were performed using the Argos Movu biometer (Argos Inc., Santa Clara, CA, USA). Parameters assessed included AL, CD, CCT, ACD, LT, PS, and keratometric values (K1, K2, and K-mean) in both patients with congenital aniridia and healthy controls. Two separate measurements were obtained for each eye. The first measurement was performed under natural, non-cycloplegic conditions with a constricted pupil. This was followed by a second measurement of the same parameters approximately 40 min after the instillation of one drop of 1% cyclopentolate (Cicloplegicedol, Laboratório Edol, Linda-a-Velha, Portugal) to induce cycloplegia.

Statistical analysis was performed using SPSS software (version 19.0; IBM, New York, NY, USA). Data from both eyes of each participant were included in the analysis. Linear mixed-effects modeling (LMM) was performed to investigate the relationships between AL, CD, CCT, K-mean, and the variables age, gender, and diagnosis. Age was entered into the models as a covariate, gender and diagnosis were specified as fixed factors, and patient ID was included as a random effect to account for correlations between measurements from both eyes of the same individual. Differences in biometric measurements obtained from eyes with congenital aniridia before and after cycloplegia were analyzed using the Wilcoxon signed-rank test. In addition, for AL, CD, CCT, and K-mean, interocular asymmetry was determined by subtracting left eye values from right eye values. Differences in interocular measurements between patients with congenital aniridia and control subjects were compared using the Mann–Whitney U test, as the variables did not follow a normal distribution. *p*-values below 0.05 were considered statistically significant.

## 3. Results

The AAK grade of the analyzed congenital aniridia subjects was as follows: Grade 0 in 4 eyes (6.78%), Grade 1 in 20 eyes (33.90%), Grade 2 in 18 eyes (30.51%), Grade 3 in 4 eyes (6.78%), and Grade 4 in 13 eyes (22.03%).

Iris malformation Grade was as follows: Grade 0 in 4 eyes (6.78%), Grade 1 in 10 eyes (16.95%), Grade 2 in 11 eyes (18.64%), Grade 3 in 4 eyes (6.78%), and Grade 4 in 23 eyes (38.98%).

In the congenital aniridia group, 41 eyes (69.49%) of 21 patients had cataracts, including anterior polar, cortical, posterior polar, nuclear, or combinations of these types. Additionally, 14 eyes (22.03%) from 7 subjects were pseudophakic, and 4 eyes (6.78%) from 3 subjects were aphakic. Among the healthy controls, all eyes had clear lenses.

**Figure 1:**
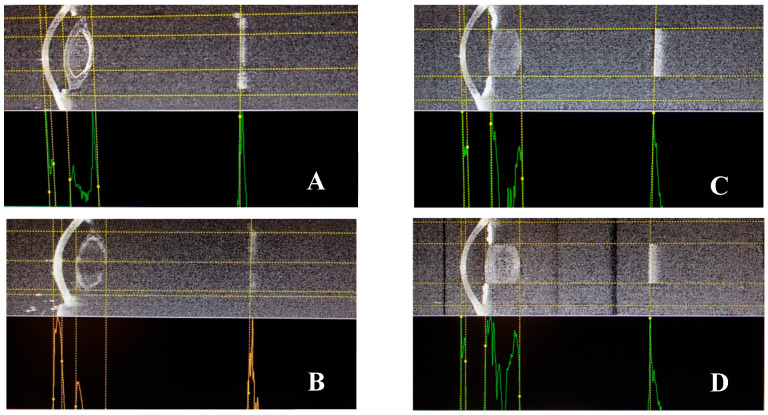
Biometry of patients with congenital aniridia (**A**,**B**), and healthy individuals (**C**,**D**), using the noncontact Argos Movu biometer (Argos Inc., Santa Clara, CA, USA). In congenital aniridia, there is a thicker cornea, thicker lens and anterior and posterior capsular cataract (**A**,**B**), which are all not visible in healthy controls with a clear lens (**C**,**D**).

The biometric measurements and the image evaluation have been performed by a single examiner (K.K.). Representative images captured using the biometer for both groups are displayed in Figure 1. Twenty-three (74.19%) of the patients with congenital aniridia exhibited varying degrees of nystagmus, which posed challenges in obtaining high-quality measurements and images.

Measurement results without and with cycloplegia in eyes with congenital aniridia and healthy controls are displayed in Table 1. The congenital aniridia and healthy subjects have been classified based on their measured CD, without and with cycloplegia. Within the congenital aniridia group, without cycloplegia, there were 5 (8.47%) eyes measured as microcornea (CD < 10 mm), and all other eyes had normal corneal diameter. In contrast, with cycloplegia, within the congenital aniridia group, all CD measurements were within the normal range. Within the healthy control group, all corneal diameter values were within the normal range, without or with cycloplegia.

Linear mixed-effects models were used to evaluate the associations between ocular parameters and age, gender, and diagnosis, while accounting for within-subject correlation (Table 2). For AL, gender was a significant predictor (β = −0.90, 95% CI: −1.74 to −0.06, *p* = 0.03), whereas age and diagnosis showed no significant associations. CD was significantly reduced in the aniridia group (β = −0.55, 95% CI: −1.01 to −0.08, *p* = 0.02), whereas age and gender were not significantly associated. CCT was strongly associated with diagnosis (β = 182.39, 95% CI: 138.08 to 226.71, *p* < 0.001), indicating substantially increased thickness in the aniridia group, while age and gender were not significant predictors. K-mean values differed significantly by diagnosis (β = −1.10, 95% CI: −2.10 to −0.10, *p* = 0.03), with lower values observed in patients with aniridia. No significant effects of age or gender were found for the K-mean. Overall, diagnosis emerged as the main determinant of CCT, CD, and K-mean, while AL was primarily influenced by gender (Table 2).

Paired comparisons of biometric parameters obtained before and after cycloplegia in eyes with congenital aniridia were performed using the Wilcoxon signed-rank test. Comparison of biometric measurements in eyes with congenital aniridia before and after cycloplegia demonstrated a significant increase in AL measurement value after cycloplegic treatment (*p* < 0.001) and significantly higher ACD measurement value (*p* = 0.01). By contrast, CD, CCT, LT, PS, K1, K2, and K-mean did not show statistically significant changes (all *p* ≥ 0.06).

Analysis using the Mann–Whitney U test demonstrated no statistically significant differences in right–left eye asymmetry between patients with congenital aniridia and control subjects across any of the assessed parameters (all *p* ≥ 0.07) (Table 3). Effect size evaluation revealed minimal group-related effects for most variables, with η^2^ values of 0.01 or less for CD and CCT. AL and K-mean showed marginally higher, yet still small, effect sizes (η^2^ = 0.04), indicating limited clinical relevance. Altogether, interocular asymmetry did not differ between the aniridia and control groups.

For AL, a significant association was observed with gender (β = −0.90, 95% CI: −1.74 to −0.06, *p* = 0.03), whereas neither age nor diagnosis showed a significant effect. CD was significantly smaller in the aniridia group (β = −0.55, 95% CI: −1.01 to −0.08, *p* = 0.02), with no significant contributions from age or gender. CCT demonstrated a strong association with diagnosis (β = 182.39, 95% CI: 138.08 to 226.71, *p* < 0.001), reflecting markedly increased thickness in the aniridia group, while age and gender were not related to this parameter. K-mean measurements varied significantly according to diagnosis (β = −1.10, 95% CI: −2.10 to −0.10, *p* = 0.03), showing lower values in patients with aniridia, while age and gender were not associated with these values.

## 4. Discussion

The present study provides an evaluation of ocular biometric characteristics in patients with congenital aniridia, highlighting the distinct influence of diagnosis, demographic factors, and cycloplegic status on biometric parameters. Using linear mixed-effects modeling to account for within-subject correlation, the diagnosis of congenital aniridia emerged as the primary determinant of CCT, CD, and K-mean, whereas AL was predominantly influenced by gender. Furthermore, the absence of marked interocular asymmetry suggested relatively symmetrical bilateral involvement in eyes with congenital aniridia. Although some reports discuss the outcomes of cataract surgery in eyes with congenital aniridia, to the best of our knowledge, only one previous study has analyzed the biometric properties of these eyes to date [20]. In this previous study, Voskresenskaya et al. specifically examined ultrasound biometric measurements, including AL, ACD, and LT [20].

Voskresenskaya et al. also did not find a significant difference in AL between 220 congenital aniridia and 344 healthy eyes [20]. In their study, measurements were performed at 11 different time points, ranging from 2 to 4 months of age to 51–60 years, using a contact Nidek US-1800 A-scanner. It should be noted that contact ultrasound biometry may induce corneal compression, potentially resulting in artificially shortened AL values. This limitation further supports the validity and reliability of the present SS-OCT-based measurements. The present study utilized a non-contact Movu biometer with a single measurement time point for each subject, and with assessments conducted both without and with cycloplegia. Consistent with the findings of Voskresenskaya et al., the diagnosis of congenital aniridia was not significantly associated with AL. In contrast, AL showed a significant relationship with gender, indicating that AL in congenital aniridia may be more strongly influenced by demographic factors than by the underlying genetic disorder itself. However, AL values were significantly higher after cycloplegia within the aniridia group compared with measurements without cycloplegia. Future studies with larger sample sizes using non-contact biometers are warranted to further validate these results. Moreover, further studies should determine reliability and acquisition success rates using the Movu biometer in patients with congenital aniridia.

Voskresenskaya et al. reported that 11% of congenital aniridia eyes in their cohort exhibited microphthalmia, while all other congenital aniridia eyes fell within the normal range [20]. In addition, LMM analysis demonstrated a significant negative effect of congenital aniridia diagnosis on CD, reflecting the underlying developmental abnormalities associated with this condition. In the present study, a small proportion of eyes (8.47%) were classified as microcorneal without cycloplegia, whereas all measurements obtained after cycloplegia were within the normal range. Non-cycloplegic measurements in congenital aniridia may be more susceptible to measurement-related factors, including reduced fixation stability, accommodation, and nystagmus. Cycloplegia may improve measurement reliability, and non-cycloplegic corneal diameter measurements should therefore be interpreted with caution. Nevertheless, this warrants further investigation.

CCT was significantly greater in eyes with congenital aniridia than in healthy controls, consistent with the presence of AAK. Similarly to this study, Wang et al. [16] and Park et al. [21] also reported increased CCT in congenital aniridia, measuring 632 ± 37 µm and 643.05 ± 37.67 µm, respectively. In contrast, the current investigation found an even higher mean CCT of 723.01 ± 156.26 µm (range: 478–1194), compared to previous studies. The markedly increased CCT observed in eyes with congenital aniridia may reflect marked structural abnormalities associated with AAK. Similarly, the reduced CD and lower K-mean values suggest altered anterior segment morphology in this patient population, likely related to disrupted ocular surface and corneal stromal maturation resulting from *PAX6*-related developmental defects and postnatal progression of AAK. These findings support the concept that congenital aniridia is characterized by widespread structural alterations extending beyond the iris.

Voskresenskaya et al. measured lens thickness in 88 eyes of 88 patients with congenital aniridia using ultrasound biometry and compared the results with age-matched controls [20]. They observed relatively high lens thickness values at 2–4 months of age, which decreased until 11–13 years. Thereafter, from the age of 13, lens thickness increased again progressively. This thickening was generally more pronounced in congenital aniridia patients than in healthy controls and reached statistical significance by 30 years of age [20]. In the current study, the mean age of subjects with congenital aniridia was 27.35 ± 17.66 years (range: 7–61), while in the healthy control group, it was 30.75 ± 23.33 years (range: 4–84). Nevertheless, a limitation of the present study is that individuals with congenital aniridia and concomitant cataract or pseudophakia could not be excluded, as the majority of included eyes belonged to these subgroups. Consequently, the independent effect of congenital aniridia on ACD and LT could not be reliably assessed in the present study.

Assessment of interocular asymmetry demonstrated no significant differences between the aniridia and control groups for any evaluated parameter. Furthermore, effect size analysis revealed consistently small group-related effects, indicating limited clinical relevance of interocular differences. This finding suggests relatively symmetrical bilateral involvement in congenital aniridia, supporting the concept of a similar developmental disruption affecting both eyes. The absence of marked asymmetry may also have practical implications for clinical monitoring and surgical planning.

Reinhard et al. [22] performed cataract surgery in 19 eyes with congenital aniridia, implanting black diaphragm aniridia intraocular lenses. They reported numerous postoperative complications, including corneal epithelial disorders, chronic endothelial cell loss, hyphema, uveitis, secondary glaucoma, and clinically significant macular edema [22]. In contrast, Wang et al. reported on 17 eyes of 10 patients with congenital aniridia who underwent cataract surgery at a mean age of 25.4 ± 14.77 years (range: 4–50 years), but their study did not document surgical complications [16]. Náray et al. conducted a large-scale study on congenital aniridia and found that previous cataract surgery was associated with a higher AAK grade and an increased prevalence of secondary glaucoma in both pseudophakic and aphakic eyes postoperatively [12]. Therefore, careful consideration is essential when planning cataract surgery in patients with congenital aniridia.

In summary, the present findings emphasize that congenital aniridia is primarily associated with alterations in corneal structure, while AL remains largely unaffected by diagnosis. The observed cycloplegia-related changes in AL highlight potential measurement-related influences that should be considered in both clinical practice and research settings. Future studies with larger cohorts and longitudinal designs are warranted to further clarify the developmental and technical factors underlying these biometric characteristics and to determine their implications for disease progression and visual outcomes. In addition, subsequent studies with larger cohorts of patients with congenital aniridia should investigate the effect of AAK grade and lens status on ocular biometric parameters.

## 5. Conclusions

*PAX6*-related congenital aniridia eyes are associated with increased CCT and reduced CD, and K-mean, while AL appears to be mainly influenced by gender. The absence of marked interocular asymmetry suggests relatively symmetrical bilateral involvement. These differences should be considered in corneal and lens surgery planning.

## Figures and Tables

**Table 1:** Biometric measurements without and with cycloplegia in congenital aniridia and healthy subjects, using the noncontact Argos Movu biometer (Argos Inc., Santa Clara, CA, USA). Axial length (AL), corneal diameter (CD), central corneal thickness (CCT), anterior chamber depth (ACD), lens thickness (LT), pupil size (PS), and keratometric data (K1, K2, and K-mean) values are displayed as mean ± standard deviation [median] (minimum–maximum). Only 25 (44.64%) of the 56 eyes with congenital aniridia were measured both without and with cycloplegia, as not all patients consented to cycloplegic examination.

	AL (mm)	CD (mm)	CCT (µm)	ACD (mm)	LT (mm)	PS (mm)	K1 (D)	K2 (D)	K-Mean (D)
***Without cycloplegia***									
**Congenital** **aniridia (n = 59)**	23.26 ± 2.85 [22.01] (20.30–32.74)	12.22 ± 1.54 [12.50] (7.87–14.58)	723.0 ± 156.25 [723.0] (478–1194)	3.20 ± 0.66 [3.0] (2.15–4.66)	3.08 ± 1.36 [3.56] (0.52–5.23)	8.33 ± 2.69 [8.92] (3.03–12.57)	40.75 ± 4.76 [41.09] (14.30–45.45)	45.19 ± 3.04 [44.46] (40.30–55.66)	42.90 ± 2.86 [42.67] (29.95–46.59)
**Controls (n = 99)**	23.41 ± 1.13 [23.36] (20.77–26.12)	12.73 ± 0.60 [12.50] (11.13–13.79)	546.24 ± 38.80 [547.0] (438–642)	3.53 ± 0.41 [3.56] (2.45–4.23)	3.97 ± 0.46 [3.81] (3.29–5.53)	5.16 ± 1.19 [5.33] (2.34–7.97)	43.34 ± 1.58 [43.31] (40.19–47.42)	44.68 ± 2.66 [44.43] (41.33–66.06)	43.88 ± 1.54 [43.71] (40.87–47.87)
***With cycloplegia***									
**Congenital** **aniridia (n = 25)**	23.59 ± 2.47 [22.95] (20.35–29.01)	13.01 ± 1.09 [13.26] (10.20–14.25)	733.52 ± 178.87 [764.0] (495–1098)	3.05 ± 0.84 [3.13] (2.20–4.32)	3.26 ± 1.27 [3.67] (0.60–5.37)	9.03 ± 2.14 [9.70] (5.05–12.05)	41.98 ± 2.05 [41.58] (39.60–45.63)	44.84 ± 2.40 [44.15] (40.93–50.58)	43.34 ± 1.87 [42.93] (40.43–46.53)
**Controls (n = 99)**	23.21 ± 2.40 [23.36] (21.27–26.12)	12.98 ± 0.49 [12.99] (11.71–14.14)	578.33 ± 125.74 [544.0] (465.0–680.10)	3.69 ± 0.39 [3.76] (2.67–4.35)	3.83 ± 0.49 [3.66] (3.28–5.29)	7.47 ± 0.94 [7.58] (4.52–9.18)	43.30 ± 1.59 [43.27] (40.25–47.56)	44.49 ± 1.57 [44.32] (41.09–48.51)	43.90 ± 1.55 [43.72] (40.70–48.03)

**Table 2:** Results of linear mixed-effects models for the analyzed ocular parameters in eyes with congenital aniridia and in healthy controls (both groups without cycloplegia) (A–D). Linear mixed-effects models were used to assess the associations between axial length (AL), corneal diameter (CD), central corneal thickness (CCT), and mean keratometry (K-mean) with age, gender, and diagnosis. Age was included as a covariate, gender and diagnosis as fixed effects, and patient ID as a random effect to account for within-subject correlation, as in most cases, both eyes of the same patient were analyzed. Values are presented as estimates, standard errors, degrees of freedom (df), t-statistics (t), *p*-values, and 95% confidence intervals. Statistical significance was defined as *p* < 0.05.

**(A) Axial length (AL)**						
***Parameter***	***Estimate***	***Standard Error***	***df***	***t***	***Significance***	***95% Confidence interval***
**Intercept**	23.83	0.42	78.02	55.86	**<0.001**	22.98 to 24.68
**Age**	0.001	0.01	80.37	0.13	0.89	−0.01 to 0.02
**Gender**	−0.90	0.42	77.48	−2.14	**0.03**	−1.74 to −0.06
**Diagnosis**	−0.08	0.42	77.50	−0.19	0.84	−0.94 to 0.76
**(B) Corneal Diameter (CD)**						
***Parameter***	***Estimate***	***Standard Error***	***df***	***t***	***Significance***	***95% Confidence interval***
**Intercept**	13.02	0.23	73.46	55.54	**<0.001**	12.55 to 13.48
**Age**	−0.009	0.005	75.65	−1.64	0.10	−0.02 to 0.001
**Gender**	−0.04	0.23	74.06	−0.21	0.82	−0.50 to 0.50
**Diagnosis**	−0.55	0.23	74.44	−2.35	**0.02**	−1.01 to −0.08
**(C) Central Corneal Thickness (CCT)**						
***Parameter***	***Estimate***	***Standard Error***	***df***	***t***	***Significance***	***95% Confidence interval***
**Intercept**	559.75	22.36	77.56	25.02	**<0.001**	515.22 to 604.29
**Age**	0.12	0.53	79.59	0.23	0.81	−0.93 to 1.18
**Gender**	−34.32	21.95	77.55	−1.56	0.12	−78.04 to 9.39
**Diagnosis**	182.39	22.25	77.68	8.19	**<0.001**	138.08 to 226.71
**(D) Mean keratometry (K-mean)**						
***Parameter***	***Estimate***	***Standard Error***	***df***	***t***	***Significance***	***95% Confidence interval***
**Intercept**	43.79	0.46	68.48	94.85	**<0.001**	42.87 to 44.71
**Age**	−0.002	0.01	71.34	−0.20	0.83	−0.02 to 0.02
**Gender**	0.36	0.46	69.04	0.77	0.44	−0.57 to 1.29
**Diagnosis**	−1.10	0.50	69.83	−2.20	**0.03**	−2.10 to 0.10

**Table 3:** Interocular Differences (Right Eye−Left Eye) in congenital aniridia and control groups (without cycloplegia). Mann–Whitney U test revealed no significant differences in right–left eye asymmetry between the aniridia and control groups for any of the evaluated parameters (all *p* ≥ 0.07). Although axial length (AL) and mean keratometry (K-mean) exhibited a tendency toward increased interocular variability in patients with aniridia, these differences did not reach statistical significance. Measures of corneal diameter (CD) and central corneal thickness (CCT) were similar between groups. Effect size analysis indicated small group effects for most parameters, with η^2^ values of 0.01 or lower for CD and CCT. AL and K-mean exhibited slightly larger, yet still small, effect sizes (η^2^ = 0.04 for both), suggesting limited practical relevance of the observed differences. Overall, these results indicate that interocular asymmetry was comparable between the aniridia and control groups, with no meaningful group-related effects detected.

	Congenital Aniridia		Controls		Mann–Whitney U Test	
Parameter	Mean ± SD	Median (IQR)	Mean ± SD	Median (IQR)	*p*-Value	η^2^
AL Δ (R–L)	−0.40 ± 1.32	−0.19 (−0.42–0.10)	−0.05 ± 0.23	0.00 (−0.15–0.09)	0.07	0.04
CD Δ (R–L)	−0.28 ± 1.19	−0.37 (−0.84–−0.07)	−0.10 ± 0.35	−0.09 (−0.23–0.06)	0.34	0.01
CCT Δ (R–L)	14.12 ± 93.42	6.00 (−10.0–64.5)	0.83 ± 9.22	1.00 (−4.0–9.0)	0.32	0.01
K-mean Δ (R–L)	−0.59 ± 2.75	0.15 (−0.74–0.91)	0.12 ± 0.44	0.14 (−0.12–0.27)	0.07	0.04

## Data Availability

The raw data supporting the conclusions of this article will be made available by the authors on request.

## Abbreviations

The following abbreviations are used in this manuscript:

AAK	Aniridia-Associated Keratopathy
ACD	Anterior Chamber Depth
AL	Axial Length
CCT	Central Corneal Thickness
CD	Corneal Diameter
LT	Lens Thickness
OCT	Optical Coherence Tomography
PAX6	Paired box 6
PCI	Partial Coherence Interferometry
PS	Pupil Size
SS-OCT	Swept-Source Optical Coherence Tomography
